# P-2229. Comparative Evaluation of Multimodal Large Language Models with Vision in Infectious Diseases Cases with Clinical Images

**DOI:** 10.1093/ofid/ofae631.2383

**Published:** 2025-01-29

**Authors:** Lemuel Non, Jacob Hodges

**Affiliations:** University of Iowa Hospitals and Clinics, Iowa city, Iowa; University of Iowa Hospitals and Clinics, Iowa city, Iowa

## Abstract

**Background:**

Real-world patient scenarios often involve a composite of text and various clinical images. We explored the performance of advanced multimodal Large Language Models (LLMs) equipped with image-to-text models (vision) in diagnosing complex infectious disease cases containing both text and clinical images.

**Figure 1:**
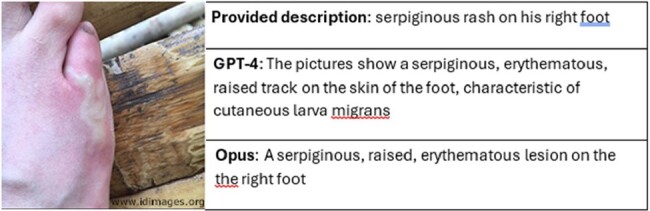
Case #19003; Right foot cutaneous lesion [Photography]. Partners’ Infectious Disease Images; Accessed on April 4, 2024 from https://www.idimages.org/images/detail/?imageid=2356

A sample image from IDImages.org showing a physical exam finding and the descriptions from the AI chatbots.

**Methods:**

We assessed the performance of two publicly accessible LLMs with vision—GPT-4 and Claude Opus—on a series of 25 complex infectious disease scenarios with 67 clinical images sourced from www.IDImages.org. Prompts were standardized for each case, including instructions to identify the diagnosis, generate differential diagnoses, and describe each image. The models' accuracy in identifying the diagnosis and including it within the differential diagnoses was evaluated using a binary scoring system (0 or 1). Image description quality was assessed using a Likert scale ranging from 0 to 2 comparing the models' outputs against provided descriptions (Figure 1). Scoring was conducted by two independent clinicians, with the final scores being computed as averages.

Figure 2

**Figure:**
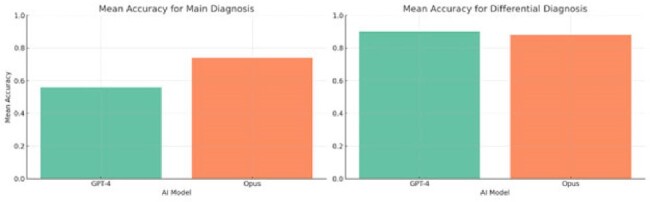

Mean scores of GPT-4 and Opus (1 denoting maximum accuracy) in correctly identifying the diagnosis (left) and in including the main diagnosis in the list of differential diagnoses (right).

**Results:**

Opus achieved a higher mean accuracy (0.74) compared to GPT-4 (0.56) in determining the correct diagnosis, with the observed difference being statistically significant (p = 0.043). Nevertheless, both models demonstrated comparably high accuracy in incorporating the primary diagnosis within the differential diagnoses, with scores of 0.90 for GPT-4 and 0.88 for Opus (Figure 2). Additionally, both models (GPT-4 average score 1.79 ± 0.43, Opus 1.75 ± 0.50) performed well in providing accurate descriptions of different clinical images. In terms of image type, they performed best in interpreting images with physical findings (GPT-4 1.92 ± 0.23, Opus 1.83 ± 0.46), followed by radiographic images (GPT-4 1.80 ± 0.41, Opus 1.83 ± 0.36), and worst in pathological specimens (GPT-4 1.61 ± 0.58, Opus 1.57 ±0.58) (Figure 3). No statistical differences were found between the two models in their overall ability to describe images or within each image category.

Figure 3

**Figure:**
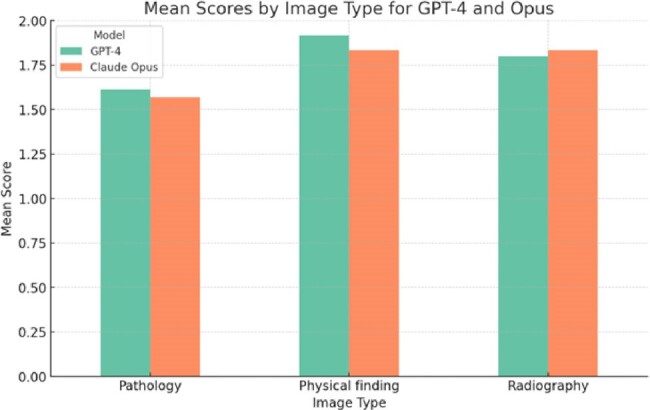

Mean accuracy scores of GPT-4 and Opus (up to 2) in interpreting clinical images grouped according to image type.

**Conclusion:**

The two models demonstrated comparable accuracy in handling complex infectious disease scenarios that integrated both texts and images, suggesting potential utility in enhancing clinical support.

**Disclosures:**

All Authors: No reported disclosures

